# Pasteurella Multocida Panophthalmitis: A Devastating Sequela of an Industrial Penetrating Injury

**DOI:** 10.7759/cureus.23962

**Published:** 2022-04-08

**Authors:** Huei Xian Chai, Yi Ni Koh, Amir Samsudin, Mei Fong Chong

**Affiliations:** 1 Ophthalmology, University of Malaya Medical Centre, Selangor, MYS; 2 Ophthalmology, Hospital Raja Permaisuri Bainun, Perak, MYS

**Keywords:** industrial injury, exogenous, endophthalmitis, panophthalmitis, pasteurella multocida

## Abstract

*Pasteurella multocida* is a rare but aggressive causative organism in panophthalmitis. It is commonly transmitted to humans through contact with cats and dogs as a result of bites or scratches. We report a rare case of panophthalmitis due to *P. multocida* following an industrial penetrating injury.

A previously healthy 40-year-old steel factory operator developed a right eye penetrating injury after being struck accidentally by a piece of iron wire which flew into his eye during work. He complained of immediate blurring of vision and severe pain in the affected eye. During history taking, he mentioned that many stray cats wandered around his workplace, although he had no direct contact with these animals. On examination, the best-corrected visual acuity was light perception in the right eye. Slit-lamp biomicroscopy revealed injected conjunctiva and a full-thickness macerated corneal laceration wound. The anterior chamber appeared shallow with the presence of lens matter. He was treated promptly with surgical repair along with intracameral and intravenous antibiotics for six hours post-trauma. His eye condition, however, deteriorated postoperatively despite aggressive treatment with further topical, intravitreal, and systemic antibiotics.

This is the first reported case of ocular *P. multocida* panophthalmitis secondary to industrial penetrating injury. Our case highlights the rapidly progressive nature of *P. multocida* infection. It should always be considered due to the very serious nature of infection as well as its resistance to standard antibiotic treatment regimens.

## Introduction

*Pasteurella multocida* is an uncommon causative pathogen in panophthalmitis, but it can cause devastating destruction of ocular tissue. It is a gram-negative coccobacillus, often co-existing as commensal in the respiratory and gastrointestinal tracts of domestic and wild animals, particularly cats and dogs. It is usually transmitted to humans through contact with animals as a result of bites or scratches. We report a rare case of panophthalmitis due to *P. multocida* in this study following an industrial penetrating injury.

This article was previously presented as a poster at the 35th Singapore-Malaysia Joint Meeting in Ophthalmology from January 17 to 19, 2020.

## Case presentation

A 40-year old gentleman who had no pre-existing medical illnesses presented to our eye clinic with a right eye penetrating injury, which occurred at his workplace on the same day of presentation. He worked as an operator in a steel manufacturing factory, where a piece of iron wire snapped off from an autowelding machine and struck him in the eye. At the time of injury, he was not wearing any eye protection. There was an immediate reduction in vision and severe pain in that right eye. During history taking, he mentioned that many stray cats wandered around his workplace, although he had no recent direct contact with them. Examination revealed best-corrected visual acuity of light perception, injected conjunctiva, and a full-thickness, 4-mm oblique macerated corneal laceration wound. The anterior chamber was shallow, with the presence of lens matter. The posterior segment could not be visualized. He was treated promptly with topical ciprofloxacin every two hours and intravenous ciprofloxacin 400 mg twice a day. The orbital x-ray did not show any intraocular foreign bodies. Lens aspiration, corneal suturing, and intracameral moxifloxacin injection were performed six hours post-injury. Intraoperatively, extensive posterior synechiae with fibrin were found in the anterior chamber. No foreign body was detected in the eye.

**Figure 1:**
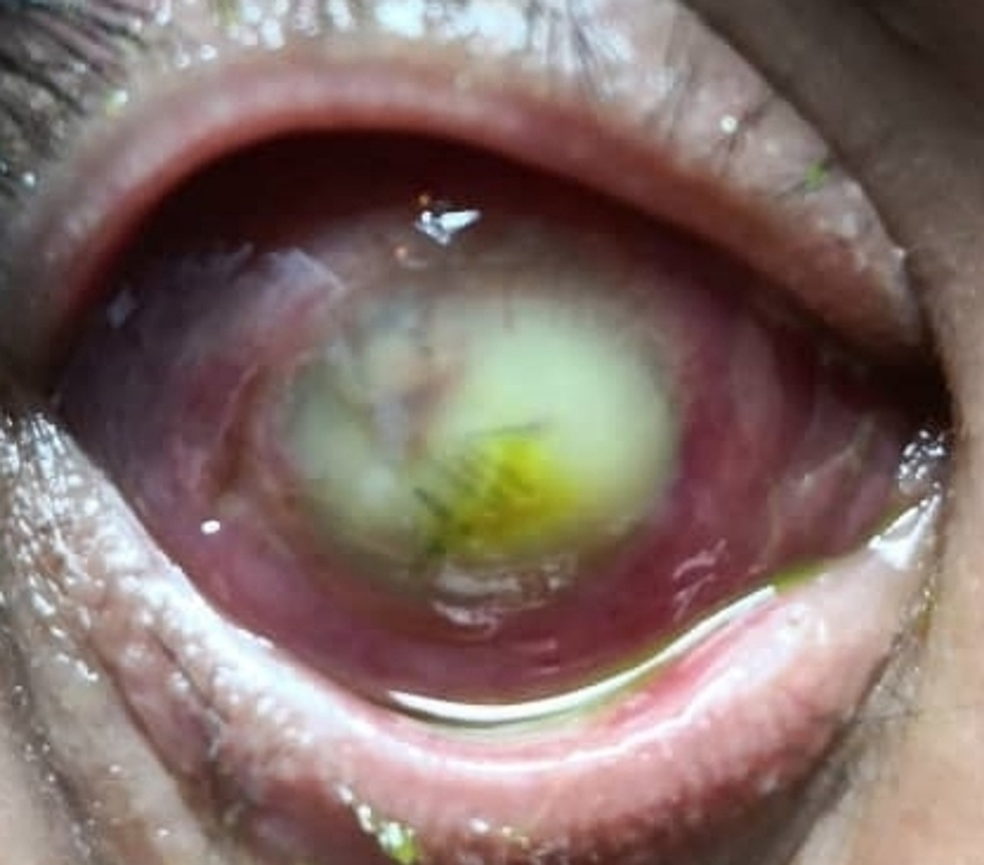
The anterior segment photo taken on postoperative day four showed conjunctiva chemosis with total hypopyon

**Figure 2:**
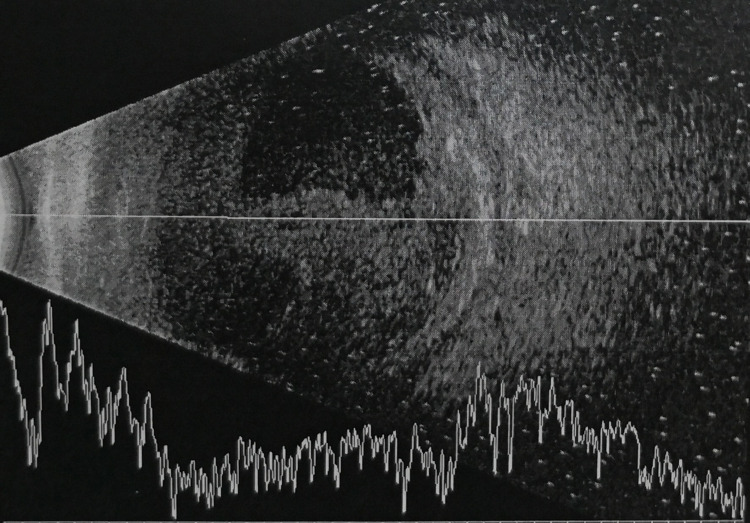
B-scan ultrasonography showed vitreous opacity and multiple loculations in the posterior chamber

**Figure 3:**
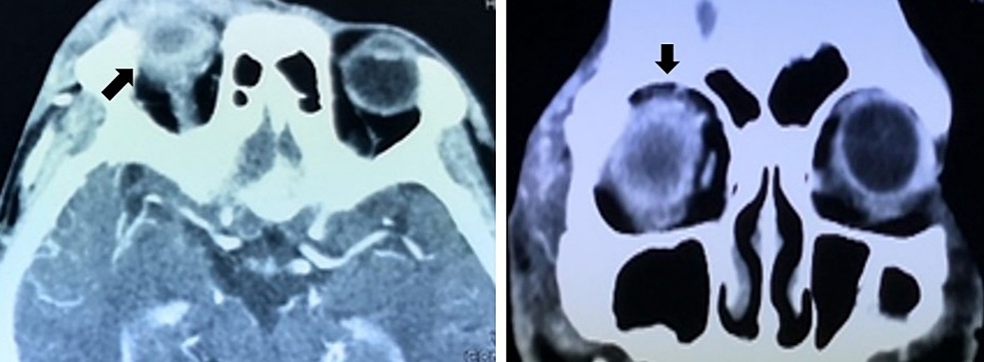
The axial and coronal views of the computed tomography scan revealed ill-defined hyperdense lesions within the right globe (arrow), with irregular septation

The patient’s eye started deteriorating on postoperative day three. Ocular motility was restricted in all directions of gaze. There was progressive eyelid swelling with severely chemosed conjunctiva and infiltration over the central cornea. The anterior chamber was filled with fibrin and hypopyon, and the fundus could not be visualized (Figure 1). B-scan ultrasonography showed vitreous opacities with multiple loculations in the posterior chamber (Figure 2). Computed tomography of the orbit revealed ill-defined hyperdense lesions within the right globe, with irregular septation (Figure 3). A diagnosis of exogenous panophthalmitis was made, and the patient was treated with intensive antimicrobial agents including topical ceftazidime 5% and topical gentamycin 1.4% hourly as well as intravenous ciprofloxacin 400 mg twice a day.

The systemic antibiotic was escalated to ceftriaxone 1 g daily and metronidazole 500 mg three times a day on day five of illness when the eye condition did not improve. Intravitreal taps were taken, and four doses of intravitreal antibiotic injections of 2 mg in 0.1 ml ceftazidime and 1 mg in 0.1 ml vancomycin were given throughout his admission. Aqueous and vitreous humor cultures both revealed the growth of *P. multocida*. Unfortunately, despite intensive antimicrobial therapy, the resolution of panophthalmitis was poor and the visual acuity ended up being no perception of light.

## Discussion

*P. multocida* is a non-motile gram-negative coccobacillus and exists as a commensal in the respiratory and gastrointestinal tracts of many domestic and wild animals. Cats and dogs have the highest carriage rates at 70%-90% and 20%-50%, respectively [1]. *Pasteurella* infection is rare but potentially sight-threatening. In humans, it is commonly caused by dog and cat bites or scratches, followed by acute onset of localized soft tissue inflammation and purulent discharge within hours. Previous reports have stated that ocular *P. multocida* infections display a wide spectrum of clinical presentations and severity, including conjunctivitis, corneal ulcers, anterior uveitis, corneal abscesses, and endophthalmitis [2]. Panophthalmitis is one of the most serious ocular complications reported from *P. multocida* infection. Other systemic involvements are respiratory tract infections, meningitis, peritonitis, endocarditis, sepsis, and bone and joint infections [3]. *P. multocida* infection without animal bites can occur, which are usually found in the patient with severe comorbidities, immune-compromised states, bacteremia, and the need for intensive care unit management [1]. It is always associated with significant mortality.

Over the years, *P. multocida-*induced panophthalmitis has rarely been reported in the literature. This organism is not a common pathogen causing panophthalmitis following open globe injury, especially when it is not associated with animal bites or scratches. Previous reports have only described systemic and localized *P. multocida* human infection involving eyes with or without evident connections with animals [2,4,5-7]. To our best knowledge, this is the first reported case of ocular *P. multocida* panophthalmitis secondary to an industrial injury. Most of the cases demonstrated poor ocular outcomes [4,5,8]. However, there have been cases that had ended with good visual outcomes after timely and appropriate management [9,10]. Mochizuki et al. reported a case of a 10-year-old girl with *P. multocida* endophthalmitis caused by a perforating ocular injury after a cat scratch where vision recovered to 20/20 [9]. The initial vision of 20/40 and early vitrectomy surgery for the patient might probably be contributing factors toward her good visual outcome in her case.

Culture and sensitivity in the setting of exogenous panophthalmitis help in the detection of pathogenic organisms, and it provides valuable information to guide the selection of the most appropriate antibiotics to be used. The treatment of choice for *P. multocida* infections has typically been with beta-lactam penicillin, second and third-generation cephalosporins, carbapenem, fluoroquinolones, and tetracyclines [11]. Awareness of the potential pathogens, early and specific antibiotic selection, with appropriate emergency treatment are the gold standards of treatment.

Our case demonstrated the rapidly devastating ocular consequence of *P. multocida* infection in a healthy young gentleman, without a history of animal bites. In our case, the organism was most likely directly inoculated inside the globe via the steel wire that had previously come into contact with stray cats. Therefore, an ocular injury with either direct or indirect exposure to an animal such as a cat or a dog should bring the clinician’s attention to *P. multocida*. The patient was treated with high-dose intravenous ceftriaxone that was adjusted according to culture and sensitivity tests along with topical ceftazidime, gentamycin, and moxifloxacin. However, despite the aggressive treatment with topical, intravitreal, and systemic antibiotics, as well as prompt surgical repair, he still had a poor visual outcome.

Industrial injury has become a norm in daily ophthalmic practice. Most of these are potentially preventable. Omar et al. reported that the majority (54.4%) of open globe injuries in Hospital Serdang, Selangor, Malaysia, were comprised of workplace injuries [12]. This case highlights the detrimental impact of industrial injury in increasing the societal burden. Preventive measures such as raising awareness of occupational safety, enforcement of workplace safety rules, and effective training programs are recommended to reduce the incidence of industrial injuries.

## Conclusions

*P. multocida* is a rare but aggressive causative organism in panophthalmitis. A literature review has highlighted the rapidly advancing and devastating outcome of ocular infection with this organism. Thus, it should always be considered as a potential causative pathogen for ocular infection, even in the absence of direct animal exposure.
